# A DNA Vaccine Formulated with Chemical Adjuvant Provides Partial Protection against Bovine Herpes Virus Infection in Cattle

**DOI:** 10.3389/fimmu.2017.00037

**Published:** 2017-01-25

**Authors:** Valeria Quattrocchi, Ivana Soria, Cecilia Ana Langellotti, Victoria Gnazzo, Mariela Gammella, Dadin P. Moore, Patricia I. Zamorano

**Affiliations:** ^1^Instituto de virología, CICVyA, INTA Castelar, Hurlingham, Buenos Aires, Argentina; ^2^CONICET, CABA, Buenos Aires, Argentina; ^3^INTA Balcarce, Balcarce, Buenos Aires, Argentina; ^4^Universidad del Salvador, Pilar, Buenos Aires, Argentina

**Keywords:** DNA, vaccine, BoHV-1, cattle, adjuvant

## Abstract

Bovine herpesvirus-1 (BoHV-1) is the causative agent of bovine infectious rhinotracheitis, an important disease worldwide. Although conventional BoHV-1 vaccines, including those based on the use of modified live virus and also inactivated vaccines, are currently used in many countries, they have several disadvantages. DNA vaccines have emerged as an attractive approach since they have the potential to induce both humoral and cellular immune response; nevertheless, it is largely known that potency of naked DNA vaccines is limited. We demonstrated previously, in the murine model, that the use of adjuvants in combination with a DNA vaccine against BoHV-1 is immunologically beneficial. In this study, we evaluate the immune response and protection against challenge elicited in bovines, by a DNA vaccine carrying the sequence of secreted version of glycoprotein D (gD) of BoHV-1 formulated with chemical adjuvants. Bovines were vaccinated with formulations containing the sequence of gD alone or in combination with adjuvants ESSAI 903110 or Montanide™ 1113101PR. After prime vaccination and two boosters, animals were challenged with infectious BoHV-1. Formulations containing adjuvants Montanide™ 1113101PR and ESSAI 903110 were both, capable of increasing humoral immune response against the virus and diminishing clinical symptoms. Nevertheless, only formulations containing adjuvant Montanide™ 1113101PR was capable of improving cellular immune response and diminishing viral excretion. To our knowledge, it is the first time that a BoHV-1 DNA vaccine is combined with adjuvants and tested in cattle. These results could be useful to design a vaccine for the control of bovine rhinotracheitis.

## Introduction

Bovine herpesvirus-1 (BoHV-1) is the etiological agent of the infectious bovine rhinotracheitis (IBR), a cattle disease with important economic consequences worldwide. This virus causes a wide variety of clinical manifestations including conjunctivitis and upper respiratory tract infection, reproductive tract lesions such as pustular vulvovaginitis/balanoposthitis, infertility, abortion in pregnant cows, and systemic infection in the newborn. BoHV-1 has been recognized as an important component of the bovine respiratory disease complex. The BoHV-1 virus infections in cattle and buffaloes are mostly mild and non-life threatening, mortality may reach 10% (1). However, the infection causes severe economic losses since it immunosuppress infected cattle predisposing animals to secondary bacterial infections, as bronchitis and pneumonitis, leading to high morbidity and mortality (2, 3). Infection also decreases milk production and produce weight loss. Since BoHV-1 is included in list B of OIE notifiable diseases (1), it imposes restrictions to the international livestock trade.

Cattle can recover from an acute uncomplicated IBR infection in 5–10 days but they are very harmful to naive herds, because BoHV-1 can undergo latency. These animals remain carriers of BoHV-1 for the rest of their lives until immunosuppressive treatments or conditions reactivate virus replication, leading to the spread of the infection (4, 5).

The virus consists of a nucleoprotein containing the genomic double stranded DNA. This center is contained into an icosahedral capsid surrounded by a lipid bilayer in which viral glycoproteins protrude (6). Viral glycoproteins are involved in several steps of viral replication (7–10). Among them, the gD is responsible for the penetration of the virus in the host cell with participation in the viral adsorption and membrane fusion (11, 12). It has cytotoxic epitopes (13, 14) and induces neutralizing antibodies (14–16). Several studies have shown the induction of antibody response against BoHV-1 in mice and cattle immunized with plasmids encoding BoHV-1 glycoprotein D (gD) (17, 18).

Bovine herpesvirus-1 uses several mechanisms to elude the host’s immune system. By spreading intracellularly, it can exist in the presence of antiviral specific antibodies (19–22). For this reason, cytotoxic T-lymphocytes (CTL) are critical for the elimination of the virus (12, 23).

Although conventional BoHV-1 vaccines, including those based on the use of modified live virus and also inactivated vaccines are currently used in many countries, they have several disadvantages; they may protect individual animals against clinical disease, but they cannot prevent either the efficient transmission of the virus or the establishment of latency. Additionally, live-attenuated vaccines are not entirely safe, because they may cause abortion, latency, and they can reactivate (24–27). For these reasons, its use is forbidden in some countries such as Argentina. Also, the vaccine strains, may downregulate the cell surface expression of major histocompatibility complex (MHC) class I molecules (28, 29), which compromises the development of CTL against not only BoHV-1 but also other viruses and intracellular pathogens. On the other hand, inactivated viral vaccines are generally poor inducers of cellular immune responses and have a relatively short duration of immunity (14).

In this context, DNA vaccines have emerged as an attractive approach for BoHV-1. DNA is taken up and expressed by cells in the tissue, and the protein is processed and presented by local antigen presenting cells (APCs). This has the benefit of intracellular expression of the antigen, which may be targeted to the class I MHC for efficient induction of cellular immune responses (30, 31). Viral surface glycoproteins gB, gC, and gD of BoHV-1 have been selected as candidate antigens in DNA immunization (32, 33). Glicoprotein D, in particular, has shown promising results in mice (34) and partial success in calves (17, 32, 33). But, since the potency of naked DNA vaccines is limited by their inability to amplify and spread *in vivo*, adjuvant incorporation could be a good option to increase the magnitude and direction of the immune response. In this regard, other authors have tested CpG oligodeoxynucleotides for their ability to enhance immune responses against viral antigens (35) and conjugation of tgD with a proteasome-dependent degradation signal in order to improve presentation *via* MHC class I (33). Recently, we have demonstrated in the murine model that certain adjuvants in combination with a DNA vaccine against BoHV-1 are capable of improving the humoral and cellular immune response against the virus (36).

Montanide-based adjuvants have been used in both veterinary and human vaccines. These formulations have been successfully commercialized and are now available for animals in a vaccine against FMDV. We reported, in the murine model and in a preliminary assay in bovines, that adjuvant Montanide 903110 (Seppic) formulated with a DNA vaccine containing the secreted version of gD is capable of inducing a better humoral and cellular response than DNA alone (37). Recently, we demonstrated that pCIgD vaccine and Montanide 1113101 adjuvant induced an increased specific cytotoxic immune response (38).

In the present study, we evaluate the immune response and protection against challenge, induced in bovines by a DNA vaccine containing the truncated, secreted version of BoHV-1 gD (36), in combination with experimental adjuvants Montanide 903110 and Montanide 1113101PR, a more concentrated version of the first one, to extend our previous studies in the murine model and to assess the protection capacity of the vaccines formulated with these adjuvants in the natural host.

## Materials and Methods

### Animals

Bovines 1- to 2-year olds, serologically negative for BoHV-1 (*n* = 20) were used. Handling and housing of animals were made in accordance with the institutional guide for the care and use of experimental animals (Council resolution number 14/07), with the approval of the Institutional Committee for Care and Use of Experimental Animals, CICUAE-INTA, Argentina. The present study did not imply animal sacrifice.

### Virus and Cells

Bovine herpesvirus-1 strain LA (Los Angeles) was propagated in Madin Darby bovine kidney (MDBK) cells grown in Eagle Minimal Medium (MEM), supplemented with 10% fetal calf serum (FCS).

For *in vitro* cell stimulation and ELISA, inactivated (15 min at 11 cm from two General Electric G875 ultraviolet bulbs) and concentrated (ultracentrifugation at 120,000 *g* for 1 h at 4°C) virus was used.

### Plasmid Construction

Construction of the pCIgD plasmid, which expresses the secreted form of BoHV-1 gD, has been previously described (36). pCIgD and pCIneo empty plasmid were amplified in transformed *Escherichia coli* DH5α and purified using anion exchange columns (Qiagen Plasmid Purification Mega Kit). They were analyzed on the basis of 260/280 absorbance ratios and restriction digests.

### Adjuvants

Montanide 903110 (named 110) and Montanide™ 1113101PR (named 101) were provided by Seppic Inc., France. Toxicological tests (Berlin test, Oral LD 50, IP LD 50, ocular irritation test, dermal irritation test, pyrogenicity) concluded the non-toxicity and favorable tolerance of these adjuvants. Montanide™ adjuvants and their components were included as authorized substances in the annex of the European Council Regulation no. 470/2009 (previously 2377/90/EC) needing no further MRL studies. On the other hand, no side effects were seen in the site of inoculation after vaccination of the animals. Montanide 903110 is a not-crosslinked charged polymer of high molecular weight dispersed in water. Montanide™ 1113101PR is the same polymer as Montanide 903110, but it has double concentration of immunostimulating complexes. They are designed to improve DNA binding capacity and transfection efficiency (Benarous, personal communication).

### Vaccine Formulations

Adjuvants were combined with 500 μg/dose DNA vaccine (pCIgD) following Seppic’s indications (ratio 24% adjuvant and 76% DNA). Additionally, pCIgD without adjuvants was also evaluated and pCIneo (500 μg/dose) was used as negative control. Vaccines were named: pCIgD-110, pCIgD-101, pCIgD, or pCIneo.

The integrity of DNA was corroborated in each vaccine before use.

### Immunization

Bovines were vaccinated intradermically (id) in the back of the ear with 1.5 ml of each vaccine (distributed in five sites). Cattle (*n* = 5/group) were vaccinated with (I) pCIgD-101, (II) pCIgD-110, (III) pCIgD, or (IV) pCIneo. The same dose was used for a booster at day 20 and 33. At 38 days post vaccination (dpv), the cellular immune response was studied. Serum samples were taken at days 0, 15, 33, 44, and 56 dpv [12 days post-challenge (dpc)]. Animals were challenged at 44 dpv.

### Measurement of Anti-BoHV-1 and Anti-gD Antibodies by ELISA

Immulon 1 plates (for BoHV-1 ELISA) or Immulon 2HB (for gD ELISA) (Dynatech Laboratories) were coated with inactivated BoHV-1 (iBoHV-1) or recombinant gD diluted in carbonate–bicarbonate buffer (pH 9.6), overnight (ON) at 4°C. Plates were blocked with phosphate-buffered saline (PBS) containing 0.05% Tween 80 and 1% ovalbumin (PBST-OVA). After washing, serum samples diluted in PBST-OVA were incubated on the plates. Negative and positive control sera were included. A rabbit anti-bovine antibody peroxidase labeled (KPL) was added. After washing, *o*-phenylenediamine-H_2_O_2_ was used as peroxidase substrate. Absorbance was recorded at 492 nm (A_492_) in a MR 5000 microplate reader (Labsystems, MN, USA). Cutoff was established as the mean A_492_ of the negative sera +2 SD. Titers were expressed as the log_10_ of the reciprocal of the highest serum dilution giving an OD higher than the cutoff.

For isotype detection, the same ELISA described for gD was used with modifications: anti-bovine IgG1, IgG2, or IgA mouse monoclonal antibodies (provided by Dr. S. Srikumaran, University of Nebraska, USA) were used, followed by incubation with anti-mouse (HRP) conjugated. Cutoff and titers were calculated as described before.

### Measurement of Neutralizing Antibodies

Neutralizing antibodies were detected by a microplate virus neutralization assay. Briefly, serum samples (1:12 dilution in DMEM) were added to 96-well cell culture plates and incubated at 37°C for 1 h with four twofold dilutions of infective BoHV-1 (10 to 80 TCID_50_). Sera-virus mixtures were then added to a MDBK monolayer and incubated at 37°C for 1 h. Then fresh DMEM 2% FCS was added. Plates were incubated for 72 h at 37°C with 5% CO_2_ and cytopathic effect (cpe) was assessed.

### Opsonophagocytosis Assay

Inactivated BoHV-1 was labeled with FITC (Sigma, St. Louis, MO, USA) as described before (39). Opsonophagocytosis of FITC-labeled BoHV-1 was analyzed by a previously described technique with minor modifications (39). Briefly, serum from vaccinated animals was mixed with FITC-labeled iBoHV-1 at 37°C for 30 min. Bovine macrophages cell line (BoMac), were then incubated for 30 min at 37°C in CO_2_ incubator with the opsonized FITC-iBoHV-1 (moi 10). Extracellular fluorescence was quenched with a 0.2-mg/ml solution of Trypan Blue. Flow cytometry was performed in a BD FacsCalibur and analyzed with CellQuest software (BD Biosciences, San José, CA, USA). Opsonophagocytosis indexes were calculated as: % of marked cells in each animal/mean % of marked cells in pCIneo group.

### Immunofluorescence Assay

Monolayers of MBDK cells were grown in chamber and infected for 24 h with reference strain BoHV-1 LA. Sera from vaccinated and unvaccinated bovines was added (30–50 μl of 1:5 dilution) at 37°C for 45 min. After two washes with PBS, anti-bovine FITC-conjugated antibody (HyClon) was added in 1:100 dilution in Evan’s blue for 45 min at 37°C.

After two washes with PBS, monolayers were seen in a fluorescence microscope.

### ALDCs Stimulation

Afferent lymph dendritic cells (ALDCs) were obtained by cannulation of pseudo afferent lymph vessels and characterized as previously described (40). Dendritic cells were incubated with culture medium (mock) or with 1 μl/ml of vaccines pCIneo, pCIgD, pCIgD-110, or pCIgD-101. After 24 h incubation, a direct surface staining was performed using monoclonal antibody DEC205-FITC (SEROTEC, UK), and an indirect surface staining was performed using monoclonal antibodies anti CD40 and MHCII (SEROTEC, UK), and anti-mouse IgG PE conjugated (Jackson laboratories, USA). Cells were fixed with 0.2% paraformaldehyde and acquired using FACScalibur cytometer and CellQuest software (BD).

### PBMCs Isolation

Blood samples were collected by venipuncture in syringes containing preservative-free heparin. PBMCs were isolated by centrifugation on Ficoll-Paque™ PLUS (density 1.077 g/ml; GE Healthcare Bio-Sciences AB) as described elsewhere (23). Cells were counted using Tripan blue.

### BoHV-1-Specific PBMCs Proliferation

PBMC suspensions obtained from each animal were labeled with carboxyfluorescein diacetate succinimidyl ester (CFSE 3 µM) for 15 min at 37°C. Cells were washed and resuspended in RPMI 1640 complete medium (RPMI 1640 10% FBS, 10 mM HEPES, 100 U/ml penicillin, 100 mg/ml streptomycin, and 50 mM 2-mercaptoethanol). CFSE-labeled PMBC were added to a 96-well plate (U-bottom) containing iBoHV-1, concanavalin A (ConA) (Sigma-Aldrich, St. Louis, MO, USA), or medium as a positive or negative proliferation control, respectively. Cells were maintained at 37°C in 5% CO_2_ atmosphere. After 5 days incubation, cells were fixed with 0.2% paraformaldehyde. Cell proliferation was analyzed by flow cytometry, using a FACSCalibur (Becton Dickinson, San Jose, CA, USA) and CellQuest software (Becton Dickinson). Proliferation indexes were calculated as [% proliferating cells stimulated with virus/% proliferating cells without stimuli]. The cutoff was established as 2.5 according to bibliographic data (41, 42).

### IFNγ Measurement

PBMCs were incubated in 96-well plates (U-bottom) with iBoHV-1, medium, or ConA for 72 h at 37°C 5% CO_2_. Culture supernatants were collected, and IFNγ was measured using a sandwich ELISA. Briefly, Immulon II plates were coated ON at 4°C with a monoclonal anti-IFNγ antibody (donated by Dr. L. Babiuk) in carbonate–bicarbonate buffer, pH 9.6. Plates were blocked with PBST–0.1% bovine serum albumin (PBST–BSA). Dilutions of samples and recombinant IFNγ standard (Serotec, UK) were added and incubated for 1 h at room temperature (RT). Plates were washed and incubated with rabbit polyclonal anti-IFNγ antibodies (produced in our lab). After 1 h incubation at RT, biotin-conjugated antibody anti-rabbit IgG was added. After 1 h incubation at RT, HRP-conjugated streptavidin (KPL, USA) was added. *O*-phenylenediamine-H_2_O_2_ was used as peroxidase substrate. The OD was determined at 492 nm. Cytokine concentrations (picograms per milliliter) were determined by interpolation in the standard curve.

### Challenge Assay

At 44 dpv, animals were challenged with BoHV-1 virus LA strain (3 × 10^6^ TCID_50_/ml) by aerosol exposition as described previously (41, 43).

At 0, 4, 5, 6, 7, 8, and 11 dpc, calves were clinically examined and rectal temperature was recorded. Clinical score after viral challenge was established according to: Grade 0 = normal; grade 1 = slight rhinitis with serous mucus with or without mild serous conjunctivitis; grade 2 = moderate/heavy rhinitis with fibrinous serous mucus with or without moderate serous conjunctivitis; grade 3 = fibrinopurulent mucus with moderate or severe conjunctivitis; grade 4 = rhinotracheitis with or without conjunctivitis (41).

Nasal swabs were obtained at 0, 4, 5, 6, 7, 8, and 11 dpc. by inserting tampons into each nostril and dipping them in MEM containing 5,000 IU/ml penicillin, 2,500 µg/ml streptomycin, and 10 µg/ml amphotericin B. For virus titration in nasal swabs, samples were serially diluted and inoculated onto MDBK cell monolayers, which were inspected for cpe. Virus titration was performed by the end point dilution method of Reed and Muench (44).

### Statistical Analysis

InfoStat program was used. ANOVA test and Dunn post ANOVA test were performed to assess if differences were significant (Control group = pCIgD for antibody titers, isotypes, proliferation, IFNγ secretion). Bonferroni post ANOVA test was used for viral shedding.

## Results

### Vaccines Induce Specific Antibodies against gD and BoHV-1

**Figure 1:**
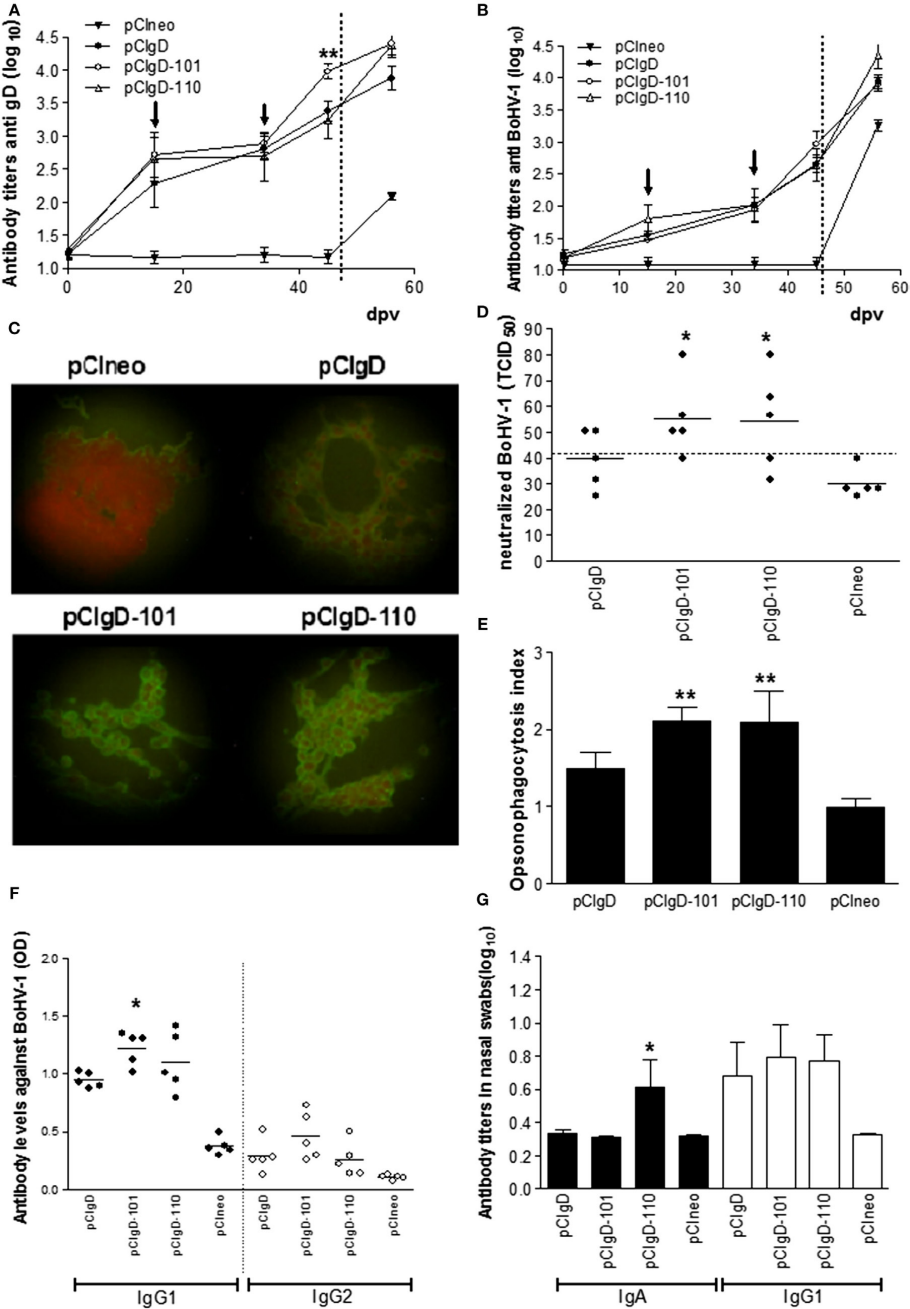
**Antibody against bovine herpesvirus (BoHV-1) elicited by vaccines**. **(A)** Antibody titers measured by ELISA using recombinant gD as antigen. **(B)** Antibody titers measured by ELISA using inactivated BoHV-1 as antigen. Dotted line represents the challenge day. Each point represents the mean titer ± SEM of the group for each date. Arrows indicate the date at which booster vaccination was performed. The cutoff was calculated as the mean level of antibodies at 0 days post vaccination (dpv) +2 SD. **(C)** Microscopy analysis of cells infected with BoHV-1 and incubated with sera from vaccinated and unvaccinated animals. One representative serum from each group is shown. **(D)** Each point represents the TCID_50_ neutralized using a 1:12 dilution of each serum at 44 dpv. The dotted line represents the cutoff point calculated as the mean level of antibodies in pCIneo group +2 SD. Solid line represents the mean TICD_50_ neutralized in each group **(E)** Opsonizing capacity of sera (from 44 dpv) measured by flow cytometry as % of BoMac cells incorporating FITC-virus after incubation with sera from vaccinated animals. Opsonizing index was calculated as: % FITC-charged cells in each group/mean % of FITC-charged cells in pCIneo group. Each bar represents the mean titer + SEM of opsonizing indexes in each group. **(F)** Isotype profile of antibodies against BoHV-1, measured by ELISA at 44 dpv. Each point represents the OD of one animal serum in a 1/50 dilution, solid line represents the mean titer of each group. Black dots represent IgG1 isotype and white dots represent IgG2 isotype. **(G)** Isotype profile of antibodies against BoHV-1, measured by ELISA at 44 dpv. Each bar represents the mean titer + SEM. Black bars represent IgGA isotype and white bars represent IgG1 isotype. Significant differences (*p < 0.5 or **p < 0.01) compared to pCI-gD group.

Analysis of sera by ELISA anti-gD (Figure 1A) showed that at 15 and 34 dpv, immunization with pCIgD, pCIgD-110, or pCIgD-101 induced specific gD antibodies. At 44 dpv, antibody levels in pCIgD-101 group were significantly higher (*p* < 0.01) than those in pCIgD and pCIgD-110 groups. Also, sera of cattle vaccinated with pCIgD, pCIgD-101, or pCIgD-110 were able to recognize the gD in the context of whole virus as detected by BoHV-1 ELISA (Figure 1B) and immunofluorescence assay (Figure 1C), although no significant differences were seen between vaccinated groups. After challenge (56 dpv), all animals seroconverted (Figures 1A,B). Neutralizing capacity of sera from groups pCIgD-101 and pCIgD-110, at 44 dpv, were slightly increased although differences were significant regarding pCIgD group (Figure 1D) and were capable of opsonizing the virus since BoMac cells incorporated significantly more FITC-virus when the virus was incubated with sera from cattle vaccinated with these vaccines (Figure 1E).

As shown in Figure 1F, at 44 dpv, pCIgD-101 group has significant higher levels of IgG1 isotype antibodies than pCIgD. IgG2 did not present significant differences among groups.

Since it is reported that antibodies in nasal mucosa can confer protection against respiratory virus infections, we studied antibodies in nasal swabs. Immunization with all formulations containing gD, at 44 dpv, induced similar titers of IgG1. IgA anti BoHV-1 was increased only in group pCIgD-110 (Figure 1G).

### Vaccines pCIgD-101 and pCIgD-110 Induce Dendritic Cells Activation *In Vitro*

**Figure 2:**
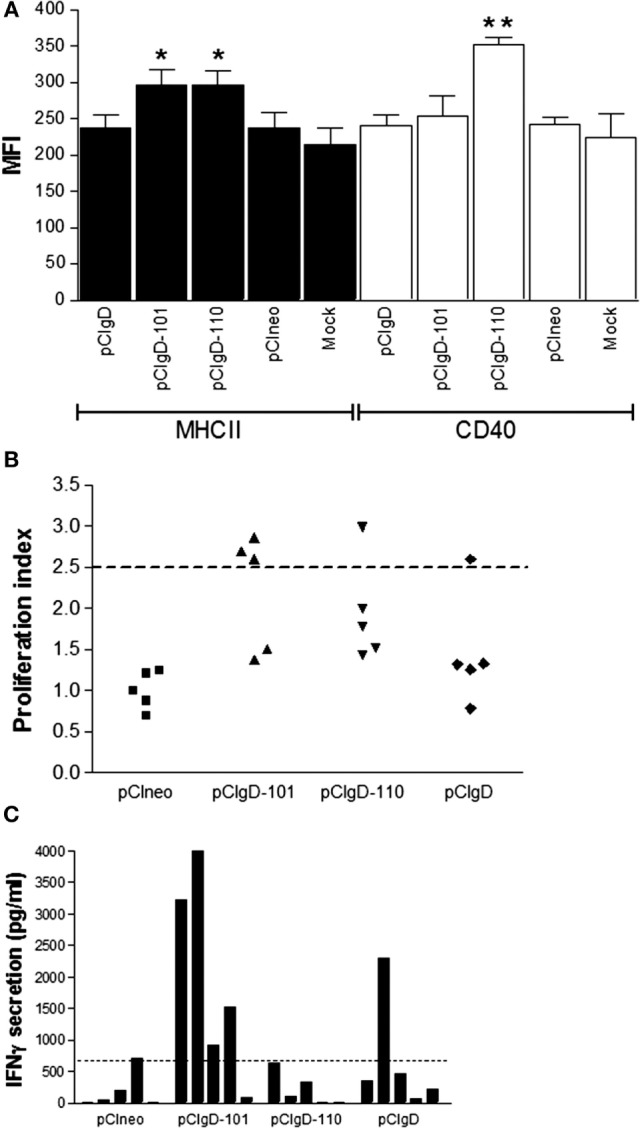
**Cellular response elicited**. **(A)** Activation of ALDCs after 24 h incubation with culture medium (mock), pCIneo, pCIgD, pCIgD-101, or pCIgD-110. Each bar represents the mean fluorescence index for five replicates plus SEM. **(B)** Specific proliferation of PBMCs taken at 44 days post vaccination (dpv) of each animal and measured by CFSE loss. Results are expressed as proliferation indexes. Proliferation indexes were calculated as [% proliferating cells stimulated with inactivated virus/% proliferating cells without stimuli]. The dotted line represents the cutoff (2.5). It was established according to bibliographic data (23, 42). **(C)** IFNγ concentrations levels measured as picogram per milliliter by ELISA in culture supernatants of PBMCs taken at 44 dpv from each animal. Dotted line represents the cutoff; it was calculated as the mean level of IFNγ at 0 dpv + 2 SD.

Dendritic cells are key initiators of antiviral responses (45) and play an important role in modulation of adaptive immune response (46). So, we studied the action of vaccines on afferent lymph dendritic cells (ALDCs). ALDCs were incubated *in vitro* with vaccines pCineo, pCIgD, pCIgD-101, and pCIgD-110. MHCII molecules were significantly upregulated (*p* < 0.05) after incubation with pCIgD-101 and pCIgD-110, and CD40 was upregulated after incubation with pCIgD-110 (*p* < 0.01) compared with pCIneo and pCIgD groups, indicating that these two vaccines can activate dendritic cells (Figure 2A).

### Cellular Immune Response Is Improved in pCIgD-101 Group

Since cellular immune response is important to deal with BoHV-1 infection, we measure specific PBMCs proliferative response and IFNγ secretion.

As shown in Figure 2B, there are more animals with a viral specific PBMCs proliferative response considered as positive (above the cutoff = 2.5) in pCIgD-101 group.

On the other hand, when PBMCs were stimulated *in vitro* with iBoHV1, most animals in this group have levels of IFNγ secretion over the cutoff point (Figure 2C).

Taken together, these results indicate that cellular response is improved by the addition of adjuvant 101 to the gD vaccine.

### Clinical Score after Challenge Is Diminished in Groups pCIgD-101 and pCIgD-110

Bovines were assayed by aerosol challenge with infective virus in order to study the protective ability of each vaccine.

Viral challenge was performed by intranasal route and bovines were monitored from 4 to 11 dpc.

A clinical score of 2 was considered as mild sickness. Every animal in pCIneo and pCIgD groups had a clinical score over 2 at some point of the assay (except for animal 517 in pCIgD group which had slight symptomatology all along the experiment). The mean duration of the symptoms was 4.6 and 2.2 days, respectively. Bovines 511, 513 (pCIneo group), and 529 (pCIgD group) presented rhinotracheitis and had to be treated with antibiotics. On the other hand, animals in groups pCIgD-101 and pCIgD-110 had lower clinical score than controls and the symptoms last for a shorter period: 2 and 1.4 days, respectively.

**Figure 3:**
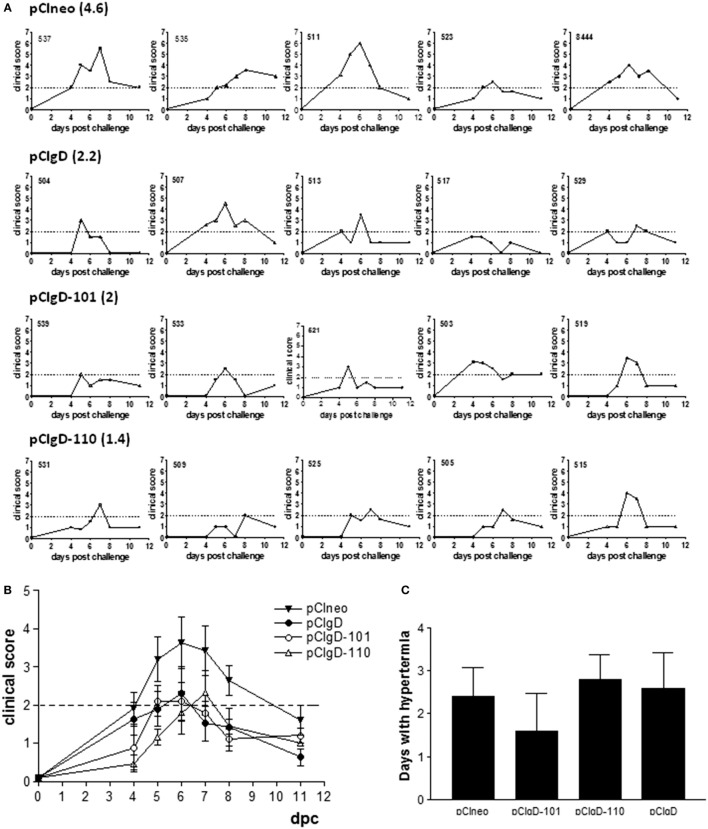
**Clinical symptoms after challenge**. Each graph represents the clinical symptoms of each animal. A clinical score was established according to the presence and severity of mucus and conjunctivitis. Dotted line represents a clinical score of 2, which was considered as mild sickness. Bold numbers indicate the mean number of days with clinical score >2 of animals vaccinated with: **(A)** pCIneo, pCIgD, pCIgD-101, pCIgD-110. **(B)** Mean number of clinical score ± SEM of each group in each time point. **(C)** Rectal temperatures. Each bar represents the mean number of days + SEM each group of animals kept temperatures above 40°C.

Despite the fact that differences were not significant, a tendency can be noticed that vaccinated cattle had lower symptomatology than pCIneo group (Figures 3A,B).

Hyperthermia tended to be lower in pCIgD-101 group than in pCIgD-110 and controls groups, although differences were not significant (Figure 3C). The exception was animal 539, which had high temperatures for 5 days, although its clinical score was low all along the experiment. It is worth to point out that, 10 dpc, all vaccinated animals present antibody titers about 4 (except in pCIneo group whose titers were about 3), indicating the induction of an anamnestic response (Figure 1B).

### Viral Excretion Is Significantly Lower in pCIgD-101 Group

**Figure 4:**
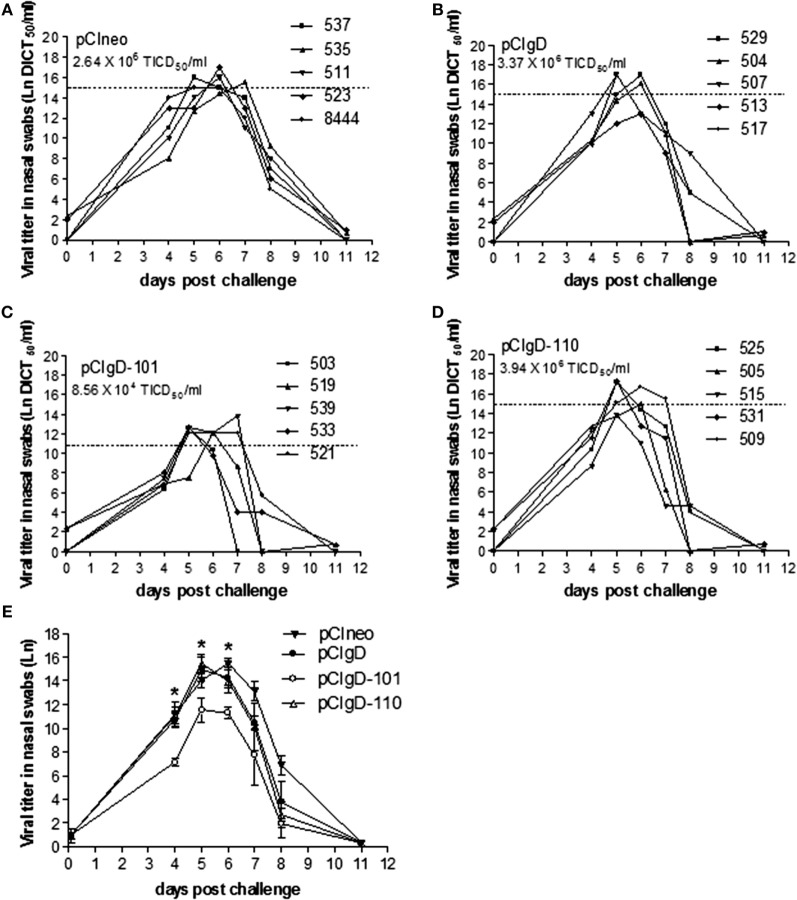
**Viral excretion after challenge**. The viral titers in each animal expressed as Ln of DICT_50_/ml are shown for groups vaccinated with: **(A)** pCIneo, **(B)** pCIgD, **(C)** pCIgD-101, and **(D)** pCIgD-110. Dotted line and numbers under the group name, represents the mean viral titer of each group during the first week after challenge. **(E)** The Ln mean viral titer of each group is shown. Significant differences (*p < 0.05).

Nasal swabs were studied for assessment of viral load in nasal secretions (Figures 4A–D).

All the bovines had detectable viremia levels. Nevertheless, the titers in group pCIgD-101 were significantly lower (*p* < 0.05) than titers in the rest of the groups at 4, 5, and 6 dpc (Figure 4E).

After the first week post-challenge, viral titers started to drop and, by 11 dpc, they became undetectable in all the groups (Figure 4).

## Discussion

It is thought that the ideal BoHV-1 vaccine should stimulate cellular and humoral arms of the immune system (47). The DNA vaccine used in the present report, a plasmid containing truncated version of gD glycoprotein plus chemical adjuvants, was widely tested in our laboratory using the murine model. Its ability to generate a specific humoral and cellular immune response against BoHV-1 is well established (36, 37). Also in mice, we demonstrated that pCIgD-101 vaccine induces an increased specific cytotoxic immune response (37). Nevertheless, the immunogenicity of pCIgD plus adjuvants in cattle was only tested in a preliminary assay (37) and protection was not reviewed. Since challenge is the most important test in order to evaluate the efficiency of a BoHV-1 vaccine (18), the present work was designed to test two candidate vaccines (pCIgD and 101 or 110 adjuvant) for protection.

It is reported that after DNA immunization truncated proteins can induce a humoral response (48). Accordingly, we demonstrated that cattle immunization with pCIgD increased the antibody levels in sera and mucosa and slightly increased the neutralizing capacity of serum and opsonizing activity, pCIgD-101 being the vaccine with the overall best humoral response at 44 dpv.

We detected a significant increase in Class II expression when DCs were incubated with pCIgD-101 and pCIgD-110. Increased expression of MHC class II leads to enhanced ability of APCs to induce T lymphocyte activation and differentiation (49). In accordance with this observation, we demonstrated that pCIgD-101 vaccine is capable of raising viral-specific PBMCs proliferation and producing IFNγ secretion in most of the vaccinated animals, indicating the induction of cellular immune response in these bovines. Also, the presence of IgG1 isotype is generally accepted as an indicator of activation of a cellular immune response (50, 51).

Taking together, these increased parameters are suggesting that a T cytotoxic response could be induced in these animals, as we observed previously in the murine model (37); nevertheless, due to the technical complications in measuring cytotoxicity in outbred animals, we could not assess the CTL response.

On the other hand, despite ALDCs stimulation induced by pCIgD-110, this vaccine do not raise IFNγ secretion and do not increase proliferation levels nor IgG1 isotype antibodies in a significative way, although in these two last cases, an incrementation tendency can be noticed.

The role of adjuvant-induced increased antigen presentation in development of adaptive immunity has not been clearly established. We hypothesize that adjuvant Montanide 1113101 PR could be facilitating DNA internalization by recruited APCs at the site of injection and thus favor gD presentation. This phenomenon was reported by Dupuis and collaborators (52), who described that MF59 adjuvant facilitated internalization of gD2 antigen from type 2 Herpes Simplex Virus by recruited APCs at the site of injection and by increasing phagocytosis in human PBMCs.

In cattle, according to the OIE manual, a vaccine is considered as protective against BoHV-1 if its capable of reduce the symptomatology to mild sickness and decrease the titer of viral shedding in 100-fold regarding control calves. Also, the excretion period must be reduced in 3 days (53). After challenge, cattle vaccinated with pCIgD-101 vaccine, had a lower clinical score and lower hyperthermia duration when compared to pCIneo group (the positive control for infection after challenge); pCIgD-110 vaccine was only able to reduce the clinical score. It is worth to note that differences in clinical score are only slight when compared to pCIgD group. Regarding viral shedding, pCIgD-101 group had significantly lower viral excretion on days 4, 5, and 6 post-challenge, compared with all groups, although only at 4 dpc this titer reach the protection criteria of 100-fold reduction regarding pCIneo group. The excretion period was similar in all groups, but the viral titers remained lower for pCIgD-101 vaccinated bovines all along the experiment. Taking together, these results indicate that protection against viral challenge achieved by the incorporation of the adjuvant 101 is only partial, which is in accordance with the slight improvements seen in humoral and cellular immunity after pCIgD-101 vaccination. On the other hand, incorporation of adjuvant 110 reduces the symptoms and viral excretion in the same way as gD alone, despite the slight improvements regarding gD that it provokes in the humoral response. We think that these results are in line with the fact that the amount of immunostimulating compounds present in adjuvant 110 is diminished regarding adjuvant 101.

Inefficient humoral immune responses have been implicated in lack of protection from BoHV-1 challenge (54), taking into account that neutralizing ability of sera is positive but low even in pCIgD-101 group, we hypothesize that this could be one of the causes of poor protection observed after challenge.

Taking into account that symptoms after viral challenge were diminished even in groups with low humoral and cellular responses, as pCIgD and pCIgD-110 groups, we conclude that the partial protection achieved against BoHV-1 in the present study involved several mechanisms that can overlap, and when one mechanism is missing, the other can work to reduce the symptoms. We do not discard the possibility that other mechanisms, independent of those studied here, were operating in the observed partial protection.

Despite the fact that inclusion of adjuvant Montanide™ EESSAI 1113101PR improved only slightly the protection against viral challenge, the present study is useful as a proof of concept to demonstrate that both, cellular and humoral arms of the immune response, are able of being stimulated by a DNA vaccine carrying the truncated version of gD formulated with a chemical adjuvant. Changes in DNA dose, the injection system of the vaccine or the addition of co-stimulatory molecules such as CD40L, or other adjuvants should be introduced, in order to improve immunity and to reduce the amount of boosters or increase the time between them.

Several studies have demonstrated the adjuvant capacity of CD40L, for both humoral and cellular immune response (55–57). Regarding other adjuvant molecules, Galectine 8 is an attractive option. Galectins are lectins that bound beta-galactosides in cellular surface, inducing proliferation and cytokine secretions among others functions (58). Galectine 8 have proved to stimulate T cell immune response *in vivo* in the murine model (59).

These results may contribute to the design of more effective vaccines against BoHV-1. To our knowledge, it is the first time that a BoHV-1 DNA vaccine is combined with chemical adjuvants and tested in cattle.

## Ethics Statement

Handling and housing of animals were made in accordance with the Institutional Committee for the use and care of experimentation animals (CICUAE). Inoculation and sampling of live animals were performed by a veterinarian. The present study did not imply animal sacrifice.

## Author Contributions

VQ: collaboration in work designing; acquisition, analysis, and interpretation of field and laboratory data; drafting; final approval of the version to be published; ensuring that questions related to the accuracy or integrity of the work were appropriately investigated and resolved. IS, CL, VG, and MG: acquisition and analysis of laboratory data for the work; critical revision for intellectual content; final approval of the version to be published; ensuring that questions related to the accuracy and integrity of laboratory work were appropriately investigated and resolved. DM: acquisition and interpretation of data related with the use of cattle; critical revision of the work for intellectual content; final approval of the version to be published; ensuring that questions related to the accuracy and integrity of field work were appropriately investigated and resolved. PZ: conception and design of the work; critical revision of the work for important intellectual content; final approval of the version to be published; ensuring that questions related to the accuracy and integrity of the work were appropriately investigated and resolved.

## Conflict of Interest Statement

The authors do not have any kind of association that might pose a conflict of interest.

## References

[1] Nandi S Kumar M Manohar M Chauhan RS Bovine herpes virus infections in cattle. Anim Health Res Rev (2009) 10:85–98. 10.1017/S1466252309990028 19558751

[2] Griebel PJ Ohmann HB Lawman MJ Babiuk LA. The interaction between bovine herpesvirus type 1 and activated bovine T lymphocytes. J Gen Virol (1990) 71(Pt 2):369–77. 10.1099/0022-1317-71-2-369 2155290

[3] Jones C Chowdhury S. Bovine herpesvirus type 1 (BHV-1) is an important cofactor in the bovine respiratory disease complex. Vet Clin North Am Food Anim Pract (2010) 26:303–21. 10.1016/j.cvfa.2010.04.007 20619186

[4] van Oirschot JT. Bovine herpesvirus 1 in semen of bulls and the risk of transmission: a brief review. Vet Q (1995) 17:29–33. 10.1080/01652176.1995.9694526 7610554

[5] Preston CM Nicholl MJ. Induction of cellular stress overcomes the requirement of herpes simplex virus type 1 for immediate-early protein ICP0 and reactivates expression from quiescent viral genomes. J Virol (2008) 82:11775–83. 10.1128/JVI.01273-08 18799580 PMC2583663

[6] Fehler F Herrmann JM Saalmüller A Mettenleiter TC Keil GM. Glycoprotein IV of bovine herpesvirus 1-expressing cell line complements and rescues a conditionally lethal viral mutant. J Virol (1992) 66:831–9. 1309917 10.1128/jvi.66.2.831-839.1992 PMC240783

[7] Okazaki K Matsuzaki T Sugahara Y Okada J Hasebe M Iwamura Y BHV-1 adsorption is mediated by the interaction of glycoprotein gIII with heparinlike moiety on the cell surface. Virology (1991) 181:666–70. 10.1016/0042-6822(91)90900-V 2014642

[8] Liang XP Babiuk LA van Drunen Littel-van den Hurk S Fitzpatrick DR Zamb TJ. Bovine herpesvirus 1 attachment to permissive cells is mediated by its major glycoproteins gI, gIII, and gIV. J Virol (1991) 65:1124–32. 1847442 10.1128/jvi.65.3.1124-1132.1991 PMC239878

[9] Brum MCS Coats C Sangena RB Doster A Jones C Chowdhury SI. Bovine herpesvirus type 1 (BoHV-1) anterograde neuronal transport from trigeminal ganglia to nose and eye requires glycoprotein E. J Neurovirol (2009) 15:196–201. 10.1080/13550280802549605 19115127

[10] Galdiero S Vitiello M D’Isanto M Falanga A Cantisani M Browne H The identification and characterization of fusogenic domains in herpes virus glycoprotein B molecules. Chembiochem (2008) 9:758–67. 10.1002/cbic.200700457 18311743

[11] Spear PG Entry of alphaherpesviruses into cells. Semin Virol (1993) 4:167–80. 10.1006/smvy.1993.1012

[12] Tikoo SK Fitzpatrick DR Babiuk LA Zamb TJ. Molecular cloning, sequencing, and expression of functional bovine herpesvirus 1 glycoprotein gIV in transfected bovine cells. J Virol (1990) 64:5132–42. 2168991 10.1128/jvi.64.10.5132-5142.1990 PMC248005

[13] Denis M Slaoui M Keil G Babiuk LA Ernst E Pastoret PP Identification of different target glycoproteins for bovine herpes virus type 1-specific cytotoxic T lymphocytes depending on the method of in vitro stimulation. Immunology (1993) 78:7–13. 8382189 PMC1421766

[14] Deshpande MS Ambagala TC Hegde NR Hariharan MJ Navaratnam M Srikumaran S. Induction of cytotoxic T-lymphocytes specific for bovine herpesvirus-1 by DNA immunization. Vaccine (2002) 20:3744–51. 10.1016/S0264-410X(02)00375-4 12399204

[15] Babiuk LA L’Italien J van Drunen Littel-van den Hurk S Zamb T Lawman JP Hughes G Protection of cattle from bovine herpesvirus type I (BHV-1) infection by immunization with individual viral glycoproteins. Virology (1987) 159:57–66. 10.1016/0042-6822(87)90347-3 3037783

[16] Oliveira SC Harms JS Rosinha GMS Rodarte RS Rech EL Splitter GA. Biolistic-mediated gene transfer using the bovine herpesvirus-1 glycoprotein D is an effective delivery system to induce neutralizing antibodies in its natural host. J Immunol Methods (2000) 245:109–18. 10.1016/S0022-1759(00)00267-2 11042288

[17] Cox GJ Zamb TJ Babiuk LA Bovine herpesvirus 1: immune responses in mice and cattle injected with plasmid DNA. J Virol (1993) 67:5664–7. 8350420 10.1128/jvi.67.9.5664-5667.1993 PMC237973

[18] Toussaint JF Coen L Letellier C Dispas M Gillet L Vanderplasschen A Genetic immunisation of cattle against bovine herpesvirus 1: glycoprotein gD confers higher protection than glycoprotein gC or tegument protein VP8. Vet Res (2005) 36:529–44. 10.1051/vetres:2005015 15955279

[19] Fuller AO Lee WC. Herpes simplex virus type 1 entry through a cascade of virus-cell interactions requires different roles of gD and gH in penetration. J Virol (1992) 66:5002–12. 1321283 10.1128/jvi.66.8.5002-5012.1992 PMC241354

[20] Kuhn JE Klaffke K Munk K Braun RW. HSV-1 gB and VZV gp-II crossreactive antibodies in human sera. Arch Virol (1990) 112:203–13. 10.1007/BF01323165 2165766

[21] Miethke A Keil GM Weiland F Mettenleiter TC. Unidirectional complementation, between glycoprotein B homologues of pseudorabies virus and bovine herpesvirus 1 is determined by the carboxy-terminal part of the molecule. J Gen Virol (1995) 76:1623–35. 10.1099/0022-1317-76-7-1623 9049369

[22] Srikumaran S Kelling CL Ambagala A Immune evasion by pathogens of bovine respiratory disease complex. Anim Health Res Rev (2007) 8:215–29. 10.1017/S1466252307001326 18218162

[23] Romera SA Hilgers LAT Puntel M Zamorano PI Alcon VL Dus Santos MJ Adjuvant effects of sulfolipo-cyclodextrin in a squalane-in-water and water-in-mineral oil emulsions for BHV-1 vaccines in cattle. Vaccine (2000) 19:132–41. 10.1016/S0264-410X(00)00104-3 10924795

[24] Whetstone CA Miller JM Seal BS Bello LJ Lawrence WC. Latency and reactivation of a thymidine kinase-negative bovine herpesvirus 1 deletion mutant. Arch Virol (1992) 122:207–14. 10.1007/BF01321129 1309641

[25] van Drunen Littel-van den Hurk S. Cell-mediated immune responses induced by BHV-1: rational vaccine design. Expert Rev Vaccines (2007) 6:369–80. 10.1586/14760584.6.3.369 17542752

[26] Muylkens B Meurens F Schynts F Farnir F Pourchet A Bardiau M Intraspecific bovine herpesvirus 1 recombinants carrying glycoprotein E deletion as a vaccine marker are virulent in cattle. J Gen Virol (2006) 87:2149–54. 10.1099/vir.0.81969-0 16847110

[27] Thiry E Muylkens B Meurens F Gogev S Thiry J Vanderplasschen A Recombination in the alphaherpesvirus bovine herpesvirus 1. Vet Microbiol (2005) 113:171–7. 10.1016/j.vetmic.2005.11.012 16343820

[28] Hariharan MJ Nataraj C Srikumaran S. Down regulation of murine MHC class I expression by bovine herpesvirus 1. Viral Immunol (1993) 6:273–84. 10.1089/vim.1993.6.273 8166934

[29] Nataraj C Eidmann S Hariharan MJ Sur JH Perry GA Srikumaran S. Bovine herpesvirus 1 downregulates the expression of bovine MHC class I molecules. Viral Immunol (1997) 10:21–34. 10.1089/vim.1997.10.21 9095529

[30] Iwasaki A Stiernholm BJ Chan AK Berinstein NL Barber BH. Enhanced CTL responses mediated by plasmid DNA immunogens encoding costimulatory molecules and cytokines. J Immunol (1997) 158:4591–601. 9144471

[31] Torres CA Iwasaki A Barber BH Robinson HL. Differential dependence on target site tissue for gene gun and intramuscular DNA immunizations. J Immunol (1997) 158:4529–32. 9144463

[32] Castrucci G Ferrari M Marchini C Salvatori D Provinciali M Tosini A Immunization against bovine herpesvirus-1 infection. Preliminary tests in calves with a DNA vaccine. Comp Immunol Microbiol Infect Dis (2004) 27:171–9. 10.1016/j.cimid.2003.09.001 15001312

[33] Petrini S Ramadori G Corradi A Borghetti P Lombardi G Villa R Evaluation of safety and efficacy of DNA vaccines against bovine herpesvirus-1 (BoHV-1) in calves. Comp Immunol Microbiol Infect Dis (2011) 34:3–10. 10.1016/j.cimid.2009.09.004 19906427

[34] Caselli E Boni M Di Luca D Salvatori D Vita A Cassai E A combined bovine herpesvirus 1 gB-gD DNA vaccine induces immune response in mice. Comp Immunol Microbiol Infect Dis (2005) 28:155–66. 10.1016/j.cimid.2004.10.001 15582691

[35] Mutwiri G Benjamin P Soita H Babiuk LA. Co-administration of polyphosphazenes with CpG oligodeoxynucleotides strongly enhances immune responses in mice immunized with hepatitis B virus surface antigen. Vaccine (2008) 26:2680–8. 10.1016/j.vaccine.2008.03.031 18430493

[36] Langellotti CA Pappalardo JS Quattrocchi V Mongini C Zamorano P. Induction of specific cytotoxic activity for bovine herpesvirus-1 by DNA immunization with different adjuvants. Antiviral Res (2011) 90:134–42. 10.1016/j.antiviral.2011.03.185 21443903

[37] Di Giacomo S Quattrocchi V Zamorano P. Use of adjuvants to enhance the immune response induced by a DNA vaccine against bovine herpesvirus-1. Viral Immunol (2015) 28:343–6. 10.1089/vim.2014.0113 26133047

[38] Gammella M Soria I Bellusci C Langellotti C Quattrocchi V Zamorano P Improvement of a DNA vaccine against BoHV-1 using chemical and molecular adjuvants. 11th Congress of the Latin American Association of Immunology (ALAI) and 10th Congress of the Asociación Colombiana de Alergia, Asma e Inmunología (ACAAI) IMMUNOCOLOMBIA Medellin (2015).

[39] Huber VC Lynch JM Bucher DJ Le J Metzger DW. Fc receptor-mediated phagocytosis makes a significant contribution to clearance of influenza virus infections. J Immunol (2001) 166:7381–8. 10.4049/jimmunol.166.12.7381 11390489

[40] Hope JC Howard CJ Prentice H Charleston B. Isolation and purification of afferent lymph dendritic cells that drain the skin of cattle. Nat Protoc (2006) 1:982–7. 10.1038/nprot.2006.125 17406334

[41] Romera SA Puntel M Quatrocchi V Zajac P Del Médico Zajac P Zamorano P Protection induced by a glycoprotein E-deleted bovine herpesvirus type 1 marker strain used either as an inactivated or live attenuated vaccine in cattle. BMC Vet Res (2014) 10:8. 10.1186/1746-6148-10-8 24401205 PMC3896737

[42] Victora GD Socorro-Silva A Volsi EC Abdallah K Lima FD Smith RB Immune response to vaccination with DNA-Hsp65 in a phase I clinical trial with head and neck cancer patients. Cancer Gene Ther (2009) 16(7):598–608. 10.1038/cgt.2009.9 19197326

[43] Del Médico Zajac MP Puntel M Zamorano PI Sadir AM Romera SA. BHV-1 vaccine induces cross-protection against BHV-5 disease in cattle. Res Vet Sci (2006) 81:327–34. 10.1016/j.rvsc.2006.01.004 16540133

[44] Reed LJ Muench H A simple method of estimating fifty percent endpoints. Am J Hyg (1938) 27:493–7. 10.1016/j.jvs.2011.05.096

[45] Ludewig B Barchiesi F Pericin M Zinkernagel RM Hengartner H Schwendener RA. In vivo antigen loading and activation of dendritic cells via a liposomal peptide vaccine mediates protective antiviral and anti-tumour immunity. Vaccine (2000) 19:23–32. 10.1016/S0264-410X(00)00163-8 10924783

[46] Palucka K Banchereau J. How dendritic cells and microbes interact to elicit or subvert protective immune responses. Curr Opin Immunol (2002) 14:420–31. 10.1016/S0952-7915(02)00365-5 12088675

[47] Manoj S Babiuk LA van Drunen Littel-van den Hurk S. Immunization with a dicistronic plasmid expressing a truncated form of bovine herpesvirus-1 glycoprotein D and the amino-terminal subunit of glycoprotein B results in reduced gB-specific immune responses. Virology (2003) 313:296–307. 10.1016/S0042-6822(03)00325-8 12951041

[48] Huang CR Lin SS Chou MY Ho CC Wang L Lee YL Demonstration of different modes of cell death upon herpes simplex virus 1 infection in different types of oral cells. Acta Virol (2005) 49:7–15. 15929393

[49] Coyle AJ Gutierrez-Ramos JC. The expanding B7 superfamily: increasing complexity in costimulatory signals regulating T cell function. Nat Immunol (2001) 2:203–9. 10.1038/85251 11224518

[50] Sin JI Kim JJ Arnold RL Shroff KE McCallus D Pachuk C IL-12 gene as a DNA vaccine adjuvant in a herpes mouse model: IL-12 enhances Th1-type CD4+ T cell-mediated protective immunity against herpes simplex virus-2 challenge. J Immunol (1999) 162:2912–21. 10072541

[51] Clerici M Shearer GM. The Th1-Th2 hypothesis of HIV infection: new insights. Immunol Today (1994) 15:575–81. 10.1016/0167-5699(94)90220-8 7848519

[52] Dupuis M McDonald DM Ott G. Distribution of adjuvant MF59 and antigen gD2 after intramuscular injection in mice. Vaccine (1999) 18:434–9. 10.1016/S0264-410X(99)00263-7 10519932

[53] Infectious pustular vulvovaginitis. Man OIE Terr Anim (2004) 1:514–26.

[54] Ioannou XP Griebel P Hecker R Babiuk LA van Drunen Littel-van den Hurk S The immunogenicity and protective efficacy of bovine herpesvirus 1 glycoprotein D plus emulsigen are increased by formulation with CpG oligodeoxynucleotides. J Virol (2002) 76:9002–10. 10.1128/JVI.76.18.9002-9010.2002 12186884 PMC136463

[55] Tripp RA Jones L Anderson LJ Brown MP. CD40 ligand (CD154) enhances the Th1 and antibody responses to respiratory syncytial virus in the BALB/c mouse. J Immunol (2000) 164:5913–21. 10.4049/jimmunol.164.11.5913 10820273

[56] Estes DM Brown WC Hirano A. CD40 ligand-dependent signaling of bovine B lymphocyte development and differentiation. Vet Immunol Immunopathol (1998) 63:15–20. 10.1016/S0165-2427(98)00077-4 9656436

[57] Haas KM Estes DM Activation of bovine B cells via surface immunoglobuIin M cross-linking or CD40 ligation results in different B-cell phenotypes. Immunology (2000) 99:272–8. 10.1046/j.1365-2567.2000.00962.x 10692047 PMC2327142

[58] Elola MT Wolfenstein-Todel C Troncoso MF Vasta GR Rabinovich GA. Galectins: matricellular glycan-binding proteins linking cell adhesion, migration, and survival. Cell Mol Life Sci (2007) 64:1679–700. 10.1007/s00018-007-7044-8 17497244 PMC11136145

[59] Tribulatti MV Figini MG Carabelli J Cattaneo V Campetella O. Redundant and antagonistic functions of galectin-1, -3, and -8 in the elicitation of T cell responses. J Immunol (2012) 188:2991–9. 10.4049/jimmunol.1102182 22357632

